# Social Cognition in Children with High-Functioning Autism Spectrum Disorder and Attention-Deficit/Hyperactivity Disorder. Associations with Executive Functions

**DOI:** 10.3389/fpsyg.2017.01035

**Published:** 2017-06-23

**Authors:** Ana Miranda, Carmen Berenguer, Belén Roselló, Inmaculada Baixauli, Carla Colomer

**Affiliations:** ^1^Departamento de Psicología Evolutiva y de la Educación, Universidad de ValenciaValencia, Spain; ^2^Departamento de Psicología Evolutiva y de la Educación, Universidad Católica de Valencia “San Vicente Mártir”Valencia, Spain; ^3^Departamento de Educación, Universitat Jaume ICastellón, Spain

**Keywords:** ADHD, ASD, facial affect recognition, theory of mind, executive functions

## Abstract

Autism spectrum disorder (ASD) and attention deficit hyperactivity disorder (ADHD) are neurodevelopmental disorders characterized by social impairments. The first objective of this study was to analyze social cognition deficits of children with ADHD, high-functioning ASD (HFASD), and typical development (TD) in their performance on explicit and applied measures of theory of mind (ToM). The second objective was to investigate the relationships between executive functions and social cognition in HFASD and ADHD. One hundred and twenty-six 7- to 11-year old children, 52 with HFASD, 35 with ADHD, and 39 with TD, performed the NEPSY-II social perception subtests. Parents estimated their children's ToM skills using the Theory of Mind Inventory (ToMI). Teacher-reported data from the Behavior Rating Inventory of Executive Function (BRIEF) were also obtained. The HFASD and ADHD groups showed worse performance on the verbal ToM task than the TD group, and only the performance of the HFASD group was significantly lower than the TD group on the contextual ToM task. Parents also estimated that the HFASD group had more difficulties on the applied ToM than the ADHD and TD groups. Furthermore, there is a different executive function-theory of mind link in the HFASD and ADHD groups: behavioral regulation processes such as inhibition and emotional control are more associated with social cognition in children with ADHD, whereas metacognitive processes such as initiation and planning have a strong association with social cognition in children with HFASD. These findings have implications for understanding social perception deficits in neurodevelopmental disorders, highlighting the need for early intervention.

## Introduction

Autism spectrum disorders (ASD) and attention deficit hyperactivity disorder (ADHD) are two disorders that stem from a disruption in the development of the brain and share the same genetic etiology, which largely explains the complexity of the impairments that characterize them (Ghirardi et al., 2017). Further evidence for common neurobiological substrates is based on the identification of similarities in the cognitive endophenotypes in individuals with these two disorders (Rommelse et al., 2011). The comparison and combination of the findings on overlapping and contrasting areas in the clinical manifestations and their possible interrelationships can help to clarify the nature of these two neurodevelopmental disorders. Two of the possible candidates for research on the cognitive profiles of ASD and ADHD are executive functioning (EF) and social cognition (Theory of Mind and affect recognition). Other possibilities such as weak central coherence or delay aversion are not appropriate for this line of research because each is related to only one of the two disorders, ASD and ADHD, respectively.

Research on social cognition is particularly valuable in understanding individual differences in social ability and exploring the social interaction impairments commonly found in both disorders. The study of the typical social cognition impairments in ASD has extended to ADHD in an attempt to identify possible similarities and divergences between the two disorders. Social cognition (SC) refers to the mental operations that underlie social interactions and involve interpersonal sensitivity in real social settings. It is a broad, complex, and multifaceted construct that reflects a wide variety of psychological processes, highlighting affect recognition (AR) and theory of mind (ToM). On the one hand, AR consists of identifying emotionally salient information in the environment, including verbal (lexical-semantic) and nonverbal (intonation, facial, visual, and body movement) cues about other people's emotions (Phillips, 2003). On the other hand, ToM is defined as the cognitive ability to attribute mental states such as thoughts, beliefs, and intentions to other people. It implies an awareness that others have minds with mental states, information, and motivations that may differ from one's own, allowing an individual to explain, manipulate, and predict behavior (Korkmaz, 2011).

The analysis of the 11 studies that have compared children and adolescents with ASD and ADHD (see Table 1) shows that these two disorders share difficulties in social cognition, both on different levels of ToM tasks and on tests of emotion recognition (Buitelaar et al., 1999; Adachi et al., 2004; Downs and Smith, 2004; Sinzig et al., 2008; Yang et al., 2009; Bühler et al., 2011; Demurie et al., 2011; Demopoulos et al., 2013; Baribeau et al., 2015; Hutchins et al., 2016) or empathic capacity (Dyck et al., 2001).

**Table 1:** Previous studies that compare social cognition skills in children and adolescents with ADHD and high-functioning ASD (*N* = 11).

**References**	**Participant: (N)**	**Total IQ**	**Theory of mind tasks**	**Results**	**Conclusions**
Adachi et al., 2004	29 ADHD MA = 9.6	96.7	Box of Marbles' task	ADHD: Success on ToM tasks = 84.6%.	Both ADHD and ASD show impairments in metaphor comprehension. ADHD: Adequate comprehension of sarcastic situations. ASD: Specific incapacity to understand sarcasm
	54 ASD MA = 9.8	96.1	Metaphoric and Sarcastic	ASD: Success on ToM task = 69.2%	
	199 TD MA = 10.0	–	Scenario test	Relationship between ToM and sarcasm	
Baribeau et al., 2015	71 ADHD MA = 9.3	100.6	Reading the mind in the eyes	Differences between ASD and ADHD in RMET Serious impairment in ASD and medium in ADHD Both groups performed worse on RMET items with a positive valence	ADHD presented a moderate alteration in social information processing, between ASD and TD. In ASD, social impairment is central. Social communication deficits and H/I traits are associated with impaired social perception abilities
	118 ASD MA = 11.6	91.4	Test child version (RMET)		
	42 ODD MA = 13.7	108.8	Social communication		
	34 TD MA = 12.5	113.1	Questionnaire (SWAN)		
Bühler et al., 2011	84 ADHD MA = 9.7	97.9	Facial emotion matching	Differences in inhibitory control (ADHD < ASD) and in the ToM performance among younger (ASD < ADHD) but not older children	Age modulates the ToM deficits in the clinical groups. Children with ASD lack ToM prerequisites and show impairments very early on, whereas children with ADHD display difficulties over the course of their development
	86 ASD MA = 10.8	105.4	Social attribution task		
	52 ASD+ADHD MA = 10.1	99.0	Battery for attention		
			Inhibition Go/NoGo		
Buitelaar et al., 1999	20 PC (9 ADHD MA = 12.3)	99.8	6 First-order ToM tasks	The two ASD groups are not distinguished on socio-cognitive performance, nor are they differentiated from the ADHD group. Other disorders showed no differences from TD	Impairments in mentalizing and emotion recognition skills are not specific to autism disorder. Individuals with ASD hardly differ from other disorders (such as ADHD) on their social comprehension in testing situations
	20 ASD MA = 12.5	102.1	4 Second-order ToM tasks		
	20 PDD MA = 10.1	98.7	Emotion recognition tasks		
	20 TD MA = 10.5	–			
Demopoulos et al., 2013	436 ADHD MA = 10.3	98.2	Social solving problems	ADHD and ASD performance was inferior to TD performance on all the measures, although the ASD group was worse than the ADHD	Children with ADHD display similar socio-cognitive impairments to those of children with ASD, although with less severity. The differences are more quantitative than qualitative
	137 ASD MA = 10.5	88.3	Affect recognition		
			Social judgment test		
Demurie et al., 2011	13 ADHD MA = 13.7	102.9	Interpersonal reactivity index	ASD presented impairments in perspective taking abilities. ADHD adolescents' performance was between ASD and TD adolescents	ToM problems are not specific to ASD. On all the measures, ASD < ADHD < TD, which would indicate a certain symptomatological *continuum*
	13 ASD MA = 14.3	101.5	Reading the mind in the eyes		
	18 TD MA = 13.8	Averege	Empathic accuracy task		
Downs and Smith, 2004	16 ADHD/ODD MA = 8.3	103.3	Cooperative Behavior (CB):Prisoner's dilemma test	ASD and TD outperformed ADHD/ODD on CB and EU.	ADHD/ODD group showed more socio-emotional and behavioral impairments than the ASD group. High-functioning ASD can develop cooperation and ToM, but experience deficits in identifying emotions and appropriate behaviors.
	10 ASD MA = 7.1	106.6	Emotional understanding-EU	ASD showed worse emotion recognition and more active/odd behavior	
	10 TD MA = 7.7	108.9	Behavioral development		
Dyck et al., 2001	35 ADHD	–	4 emotion recognition scales (Empathic abilities, EA)	Autism groups presented alterations in ToM and empathic abilities	Deficits in empathic capacity are not specific to the autism spectrum. Empathy is not independent from intelligence, so that both dimensions are necessary to discriminate ASD from other disorders
	48 ASD		ToM. Strange stories test	ADHD group showed deficits in empathy, but not in ToM	
	48 Other disorders		Wechsler intelligence scale		
	36 TD MA = 12.09				
Hutchins et al., 2016	29 ADHD MA = 9	Average IQ range	9 Measures of explicit ToM	ASD: Worse performance than children with ADHD and TD on all measures	Complexity in assessment is critical to distinguish the groups. ADHD fail to apply ToM abilities in the real world. ASD presented global ToM difficulties that seem attributable to a deeper meta-representational deficit
	67 ASD MA = 9.7				
	49 TD MA = 8.8		TOMI Inventory (measure of applied ToM competence)	ADHD: Equal performance to TD on explicit ToM, but lower on TOMI	
Sinzig et al., 2008	30 ADHD MA = 12.7	100	Facial affect recognition (Frankfurt Test). Inhibition Attention, Set-Shifting	ADHD groups performed significantly worse than the control group on Affect Recognition	The facial affect recognition ability is reduced in children suffering from ADHD symptoms, both in autistic and pure ADHD children
	21 ASD+ADHD	102			
	19 ASD MA = 12.6	111			
	29 TD MA = 12.8	109			
Yang et al., 2009	26 ADHD MA = 8.2	109.9	ToM 1. Appearance-reality	Children with ASD performed worse than ADHD and TD children on ToM tasks	Significant relationship between ToM and inhibitory control after partialling out IQ. ToM deficit with intact EF is unlikely
	20 ASD MA = 8.0	96.6	ToM 2. Unexpected location	ADHD performed similarly to the TD group	
	30 TD MA = 8.1	118.2		EF: Stroop, Wisconsin Test	

MA, Mean age; ADHD, Attention deficit hyperactivity disorder; ASD, Autism spectrum disorder; TD, Typical development; ODD, Oppositional defiant disorder; PDD, Pervasive developmental disorder; PC, Psychiatric control group;

*EF, Executive functions*.

With regard to the severity of the dysfunction in ToM and AR abilities, the studies indicate that the differences between ASD and ADHD are generally more quantitative than qualitative. People with ASD are affected more severely, producing a symptomatological continuity where ADHD holds an intermediate position between TD and ASD (Demurie et al., 2011; Demopoulos et al., 2013; Baribeau et al., 2015). In fact, individuals with ADHD, compared to ASD, seem to perform similarly to the TD group on appearance-reality tasks (Yang et al., 2009). However, some studies diverge from the general tendency, revealing that children with ADHD could not be differentiated from children with high-functioning autism on their performance on ToM tasks, especially on second-order mentalizing skills (Buitelaar et al., 1999).

In addition, some findings have shown a negative effect on AR when ADHD symptoms are associated with ASD. When comparing the facial AR skills of groups of children and adolescents with ASD, ADHD, ASD+ADHD, and TD, the findings showed that the groups with ADHD symptoms had more AR difficulties than the groups with only ASD or TD (Sinzig et al., 2008). Likewise, when ADHD is accompanied by oppositional-defiant disorder (ODD), there is an additional impairment in ToM development, so that the ADHD/ODD group showed more socio-emotional and behavioral impairment than the ASD group, which exhibited cooperation and ToM skills (Downs and Smith, 2004). Along with comorbidity, the types of ASD and ADHD are factors that should also be taken into account when evaluating the results. For example, one study that included only children with ADHD with a predominance of inattention did not find ToM impairments in the ADHD group, although this group showed less empathic ability. The children with severe ASD experienced pronounced deficits in ToM skills and the capacity for empathy. By contrast, the group with high-functioning autism (with Asperger's syndrome) showed a similar profile to the group with no psychopathologies (Dyck et al., 2001).

The developmental stage is another aspect to consider in ToM skills because the development of mentalizing skills seems to follow a different course in ASD and ADHD. Although, there is a lack of studies with a longitudinal design, using a cross-sectional design (Bühler et al., 2011), a significant difference in facial emotion recognition was observed between the youngest ASD and ADHD groups (ASD < ADHD), and this difference did not occur in the older groups. Therefore, social cognition impairments occur later in ADHD than in ASD, probably depending on experiences in the family circle and with classmates.

There is a noteworthy influence of the procedure used to measure the mentalist skills on the results, as reflected in the study by Hutchins et al. (2016). On all the measures, both on the battery that rated explicit ToM knowledge and on the TOMI inventory, focused on applying ToM to daily life situations, the ASD group had worse performance than the other groups. However, the ADHD group achieved higher performance on the explicit tasks, that is, on the laboratory measures. In fact, no differences were found between the ADHD and neurotypical groups on the composite score on the ToM battery (explicit knowledge), whereas on the TOMI inventory scores, the ADHD group showed significantly lower performance levels than the TD group. The authors argue that this result may be due to the fact that the application of ToM skills in daily life requires a greater number of executive functioning (EF) resources (inhibition, working memory, attention). Another possible explanation is related to the quality of the EF resources, as the applied ToM would imply a greater involvement of the “Hot” EF, which are activated to address problems that require affect and motivation regulation. This circumstance would also justify the intermediate performance of the ADHD group on the Early and Basic Subscales and their impaired performance on the Advanced subscale of the ToMI: “It may be that the Early, Basic, and Advanced Subscales require variable thresholds of EF for success” (Hutchins et al., 2016, p. 104).

The concept of EF refers to skills that make it possible to maintain an adequate response pattern to reach a future goal and dynamically self-regulate one's behavior (Goldstein et al., 2013). It includes a broad spectrum of higher mental processes that are grounded in prefrontal and thalamic-reticular areas of the brain. Their function is to direct the thoughts, actions, and emotions, and, therefore, they play a decisive role in socio-personal adaptation (Barkley, 2011; Diamond, 2013).

Poor executive functions are an established deficit of two of the most common neurodevelopmental disorders, ADHD (Barkley, 1997) and ASD (Lai et al., 2017). “ase provide the city name for”). In fact, EF deficits are the neuropsychological traits common to both disorders that have received the most support in the literature (Rommelse et al., 2011), although findings related to the specific subdomains of the deficit are not completely consistent. On the one hand, some studies suggest that the EF differences between ASD and ADHD are more quantitative than qualitative, so that it is difficult to use executive measures to differentiate the specific domains affected in each of these disorders. On the other hand, a large number of studies have provided evidence for the dissociation of the EF deficits in ADHD and ASD. Children with ASD would mostly show an alteration in planning, whereas the deficits of children with ADHD would affect inhibition and verbal working memory (Gargaro et al., 2011; Miranda-Casas et al., 2013; Craig et al., 2016).

Problems in inhibition, information recall, flexibility, and the ability to monitor, plan, and initiate socially appropriate responses—all aspects of EF—may contribute to the social impairments experienced by children with ADHD and children with ASD. A large body of evidence supports the associations between the EF and ToM in typical development (Carlson et al., 2004; Hughes and Ensor, 2007). However, few studies have examined the interesting interplay between EF and social cognition in children with neurodevelopmental disorders such as ASD and ADHD. In the case of ADHD, the findings point out that the inhibitory problems of children with ADHD impede the representation of mental states and ToM skills. In other words, ToM itself remains intact, but there is a failure to express this ability (Papadopoulos et al., 2005; Sodian and Hulsken, 2005). Supporting this view, Mary et al. (2016) hypothesize that ToM dysfunctions in children with ADHD could be due to attentional or executive deficits. They found that children with ADHD performed worse than neurotypical children on all the EF tests (inhibition, planning, attention) and two higher-order ToM tasks. The most interesting result was that, controlling inhibition and attention, the performance of children with ADHD was similar to that of children with typical development. By contrast, controlling the ToM scores did not normalize their performance on inhibition and attention tasks, indicating that EF and attention deficits were determining the ToM failures in children with ADHD.

The literature also supports the critical role of EF in the ToM skills of children with ASD. Specifically, cognitive flexibility and shifting skills have shown a significant correlation with ToM tasks that require children to change from their own perspective to that of others (Zelazo et al., 2002; Pellicano, 2007). Furthermore, cognitive shifting predicts ASD preschoolers' capacity to shift between their own beliefs and a false belief presented (Kimhi et al., 2014). Likewise, in a longitudinal study conducted over a 3-year period, early EF and central coherence skills predicted developmental changes in ToM skills, regardless of age, language, and nonverbal intelligence (Pellicano, 2010). An important and unexplored question is related to the functional link between ToM and EF in ASD and ADHD. To the best of our knowledge, in the only study that analyzed this question, carried out by Yang et al. (2009), the data from the correlational analyses showed that only inhibition was significantly related to ToM performance after partialling out non-verbal intelligence. Yang et al. (2009) applied laboratory neuropsychological tasks to measure EF. However, given the differences between the demands of a structured evaluation scenario and those of the real world, it is essential to collect the ratings of a set of metacognitive, behavioral, and emotional abilities in daily life by informants who know the subject well. Two recent studies have provided interesting findings. On one hand, it has been found that metacognitive executive processes (i.e., initiation, working memory, planning, organization, and monitoring) predicted social function only in children with ASD and not in TD children (Leung et al., 2016). On the other hand, teachers' ratings of poor initiation, working memory, and planning and organizational skills have been related to more time engaged in solitary play, suggesting that metacognitive skills such as initiation, working memory, and planning and organization are associated with social functioning in children with autism (Freeman et al., 2017).

In summary, a growing body of research supports the existence of social cognition impairments in children with ASD and children with ADHD and, to a lesser extent, differential patterns across these disorders, with more significant social perception deficits in ASD and milder deficits in ADHD. However, other findings provide relatively mixed evidence, possibly due to factors such as the broad age range of the participants, the developmental stage, the nature of the ToM measurement procedure itself (explicit or applied), group differences in intelligence, and the diagnosis of the type of presentation of the ADHD participants. Thus, one important area for future research is the continued investigation of these two neurodevelopmental disorders. This study builds on the previous one, focusing on the analysis of similarities and differences between the implicit and explicit ToM systems and possible links between ToM and EF in children with HFASD and children with ADHD. In order to obtain reliable conclusions, critical factors have been controlled, specifically the intelligence level (IQ) and verbal ability, given the degree of overlap between IQ and EF and the relationships between verbal ability and the capacity to represent mental states (Happé, 1995).

Considering the significance of these findings about similarities and differences between ASD and ADHD in the conceptualization of these two disorders, the first objective of this study is to compare the AR and ToM abilities, both explicit (laboratory tasks) and applied (parent ratings in real life—ToMI), in children with HFASD, ADHD, and TD, matched on chronological age, mental age, and vocabulary. Children with HFASD are expected to have worse performance on all the measures of social cognition than the children with TD. Children with ADHD are expected to show a more variable performance, depending on the type of measure, and occupy an intermediate position between the children with HFASD and the children with TD.

The second objective consists of exploring in the two clinical groups, children with HFASD and children with ADHD, the interplay between AR and ToM skills and “everyday” components of EF measured by the BRIEF. Based on the limited literature examining the relationship between executive and social functioning in children with ASD and children with ADHD, we predict that the EF components that have a significant relationship with the ToM in both disorders will differ. Specifically, we expect that EF behavioral regulation index subdomains, a characteristic deficiency seen in individuals with ADHD (Barkley, 1997; Mary et al., 2016), will maintain a more significant relationship with AR, ToM tasks, and ToMI total scores in this group. We also expect that the EF metacognitive index subdomains in the group with ASD will show a higher relationship with the overall indicators of AR, ToM tasks, and the ToMI performance (Leung et al., 2016; Freeman et al., 2017).

## Materials and methods

### Participants

Participants in this study were 126 children from 7- to 11-years old, distributed in three groups: a group with HFASD (*n* = 52), a group with ADHD (*n* = 35), and a group with TD (*n* = 39). Mean age of children with HFASD, *M* = 8.59, *SD* = 1.38; children with ADHD, *M* = 9.14 *SD* = 1.41; children with TD, *M* = 8.46, *SD* = 1.27. The decision on the age range was motivated by developmental reasons (coinciding with the phase of the development of concrete operational stage) and due to the importance of Primary Education in the Spanish education system that coincides with this developmental period. All the participants had an overall IQ equal to or above 80, measured with the K-BIT (Kaufman and Kaufman, 2000): Children with HFASD, *M* = 101.42, *SD* = 12.65; children with ADHD, *M* = 99.03, *SD* = 9.87; children with TD, *M* = 102.21, *SD* = 8.70. The three groups were balanced on age, *F*_(2, 123)_ = 2.61, *p* = 0.078, total IQ, *F*_(2, 123)_ = 0.865, *p* = 0.423 and semantic language level, assessed with the vocabulary subtest of the WISC-IV (Wechsler, 2003) *F*_(2, 123)_ = 1.03, *p* = 0.350. In addition, 91.42% of the HFASD group and 94.3% of the ADHD group were male.

The children in the two clinical groups had received a diagnosis of HFASD or ADHD in the Child Neurology and Child Psychiatry Services of hospitals and medical centers in the provinces of Valencia and Castellón (Valencian Community, Spain). The clinical diagnoses of HFASD and ADHD were confirmed before applying the battery of tests selected for the present study. Specifically, to confirm the HFASD diagnosis, strict cut-off points recommended for the Social Communication Questionnaire (SCQ; Rutter et al., 2003) and the revised Autism Diagnostic Interview (ADI-R; Rutter et al., 2006) were applied. These two instruments were administered to the parents by a clinical psychologist from the research team who had been accredited for their application.

The ADHD diagnosis was also confirmed through interviews with parents and teachers, who provided information about the 18 ADHD criteria from the DSM-5 (American Psychiatric Association, 2013), rating the severity of each item from 0 to 3. The requirements were the presence of at least six inattention symptoms and/or another six hyperactivity/impulsivity symptoms, persistence of the symptoms for at least 1 year, and their clear interference in the subject's daily functioning. Another requirement was a T score equal to or greater than 63 on the scales related to the ADHD diagnosis on Conners' Rating Scales—Revised, parents version (CPRS-R:L; Conners, 2001). The majority of the subjects in this group, 77.14%, showed a combined ADHD presentation, whereas 22.86% met the criteria for a presentation with a predominance of inattention. As would be expected, the rate of behavior problems (identified with the Strengths and Difficulties Questionnaire, SDQ, filled out by the parents; Goodman, 1997) in our ADHD sample was 40%.

Regarding the school modality, three children with HFASD (5.8%) and two children with ADHD (5.7%) were attending school in regular classrooms full time; 29 children with HFASD (55.7%) and 33 with ADHD (94.3%) attended regular classrooms but received educational support for their specific needs in the school; and finally, 20 children with HFASD (38.5%) were placed in the Communication and Language classrooms modality.

Participants with TD were all attending school in regular classrooms in the same schools where the children with ADHD and HFASD attended. They had no history of psychopathology or referral to children's mental health units (USMI), according to the information from the school records, and they did not meet the DSM 5 criteria for ADHD or HFASD on the screening carried out by SCQ before beginning the evaluation.

The exclusion criteria for the children who participated in the study were evaluated through an extensive prior anamnesis carried out with the families. They included severe or genetic neurological diseases, brain lesions, psychosis, visual, auditory, motor, or sensorial deficits, and an intelligence coefficient <80. The administration of psycho-drugs was only an exclusion criterion for the TD group. By contrast, 32.7% of the children in the HFASD sample were taking antipsychotics (mainly Risperidone), and 71.4% of the children in the ADHD sample were receiving psychostimulants, generally time-released (mainly Methylphenidate).

### Procedure

The study was approved by the Ethics Committee of the University of Valencia (Declaration of Helsinki in the Convention of the Council of Europe, 1964). It obtained the approval of the Consellería de Educación de la Generalitat Valenciana to locate children who had received a previous diagnosis of HFASD or ADHD by professionals in specialized childhood mental health services. The families were personally told about the aims of our research and asked for their informed consent. Oral permission from the children and written informed consent from their parents were obtained before beginning the evaluation, which took place in the schools in which they were enrolled.

The intelligence test and the two tests from the social perception domain were administered to all the children individually by trained examiners in prepared classrooms in the different schools. The parents provided information about their children's ToM skills in daily life contexts, and the teachers-tutors filled out the questionnaire selected to assess the EF.

### Measures

#### Affect recognition-AR

The subtest from the social perception domain of the NEPSY-II battery (A Developmental Neuropsychological Assessment Battery, Korkman et al., 2007) was applied.

It consists of four different tasks, and its purpose is to assess the ability to recognize the six basic emotions: happiness, sadness, fear, anger, disgust, and neutral, in photographs of children's faces. On the first and second tasks, the child is shown three photographs and asked to select two photographs of faces that express the same emotion. On the third task, the child must select one of the four faces that show the same emotion as the photograph that appears in the upper part. On the final task, the child is shown a face for 5 s and immediately asked to point to the two photographs, out of six, that show the same emotion as the face shown previously. The total score ranges between 1 and 25 in children aged 6 or younger and between 1 and 36 in children aged 7 or older, with higher scores reflecting a greater ability to match the same emotions.

#### Theory of mind abilities (ToM)

In our evaluation of the ToM, we accept the distinction made by Hutchins et al. (2016) between explicit ToM competence, defined as conceptual, operative, and logical knowledge, and applied knowledge, which involves the capacity to display ToM knowledge when facing everyday real-life dilemmas.

##### Explicit ToM knowledge

To evaluate explicit ToM knowledge, we used the Theory of Mind subtest included in the social perception domain of the NEPSY-II battery (A Developmental Neuropsychological Assessment Battery, Korkman et al., 2007). This test was designed to evaluate the capacity to comprehend mental functions such as beliefs, intentions, deception, and emotions, among others. The purpose, therefore, is to assess the capacity to understand that others have thoughts and feelings that might be different from one's own. Likewise, it aims to measure the capacity to understand the relationship between the emotions and the social context, and identify the appropriate emotion in specific social contexts. It consists of two tasks. The verbal tasks consist of showing the child an image and describing a situation related to that image. Next, the child is asked a question that requires him/her to comprehend another person's point of view. The tasks combine first- and second-order false belief questions, double deception tasks, and comprehension of figurative language.

The verbal tasks also include questions based on verbal scenarios with no pictorial support. An example for children from 7 to 11 years old would be the following: “the children in the class of Judith, their teacher, were decorating Christmas cards. Since it was almost time for recess, Judith said: “children hurry up!” What did she mean? There are also two items that measure verbal imitation and gesture skills, which are considered a basic ToM factor. The contextual task measures the child's capacity to relate an emotion to the social context. It is composed of 6 items that show images of a girl in different social contexts. From four photographs of the girl's face with different emotions, the child is asked to identify the one that has the same emotion as the girl in the picture. The child can respond by pointing to the photographs, and no verbal responses are necessary.

##### Applied ToM knowledge

Theory of Mind Inventory (ToMI, Hutchins et al., 2014. Spanish adaptation: Pujals et al., 2016). The ToMI inventory for parents or caregivers is composed of 42 items, and its objective is to address the breadth and complexity of mentalist skills. The items are grouped in 3 subscales, and they offer a general average score. The early subscale (ToMI-E) is composed of 7 items and assesses ToM skills that begin to develop in the first stages of childhood, such as social references and understanding basic emotions (e.g., “my child understands that when I show fear, the situation is unsafe or dangerous,” or “my child recognizes when others are happy”). The basic subscale (ToMI-B) includes 19 items that encompass ToM characteristics typical of children beginning the educational stage, such as the basic meta-representations and the distinction between physical and mental representations [e.g., “If I showed my child a cereal box filled with cookies and asked him/her “What would someone who has not looked inside think is in the box?” My child would say that another person would think there was cereal in the box” (false belief)]. Finally, the advanced subscale (ToMI-A) is composed of 16 items that assess more mature aspects of the ToM that develop between 5 and 8 years old, such as second-order inferences and making complex social judgments (e.g., “My child understands the difference between a friend teasing in a nice way and a bully making fun of someone in a mean way”). Each item is rated from 0 to 20, from “Definitely not” to “Definitely,” with a mid-point of “Undecided.” Responses are made with a vertical mark on a continuous line, indicating the score that best reflects the degree of agreement with each statement presented. High scores show the perception of good ToM competence.

The ToMI inventory has been extensively validated, and it has shown test-retest reliability, internal consistency, and criterion validity for samples with typical development and ASD. It has also shown excellent sensitivity (0.9) and specificity (0.9) when used to examine children with ASD, although its purpose is not to perform a differential diagnosis (Hutchins et al., 2012).

#### Executive functioning

##### Behavior rating inventory of executive function; (BRIEF, Gioia et al., 2000)

The teacher version of the questionnaire was applied to assess the child's EF through the teacher's observation of his/her behavior in the school context. It consists of 86 items rated on a Likert-type scale with three levels (never, sometimes, often). The items are grouped in 8 scales: Inhibit (“Interrupts others”); Shift (“Becomes upset with new situations”); Emotional control (“Overreacts to small problems”); Initiate (“Is not a self-starter”); Working Memory (“Has a short attention span”); Plan/Organize (does not finish long-term projects); Organization of materials (“Leaves playroom a mess”); and Monitor (“Does not check work for mistakes”).

The scales make up two indexes. The Behavioral Regulation Index (BRI) is composed of the Inhibit, Shift, and Emotional Control scales, assessing the child's capacity to make cognitive changes and adjust his/her emotions and behavior through appropriate inhibitory control. The Metacognition Index (MI) is composed of the Initiate, Working Memory, Plan/Organize, Organization of materials, and Monitor scales. This index reflects the child's ability to initiate, plan, organize, self-monitor, and maintain working memory. It could be interpreted as the ability to self-manage cognitive tasks and supervise their performance. This index is related to the capacity to actively solve problems in a variety of contexts.

The direct scores can be transformed into T-scores, with higher scores indicating worse EF. The questionnaire's reliability and validity have been adequately demonstrated (Gioia et al., 2002; García Fernández et al., 2014).

## Results

### Statistical analyses

Statistical analyses were performed using the software Statistical Package for the Social Sciences (SPSS), version 22.00 (SPSS Inc., Chicago, IL USA). Multivariate Analysis of Variance (MANOVA) was conducted to analyze differences between the HFASD, ADHD, and TD groups on the AF and ToM (explicit knowledge) subscales of the NEPSY-II and the ToMI inventory (applied knowledge). For the additional ANOVAs, the level of significance was set at *p* < 0.006 after applying the Bonferroni correction, and η^2^_*p*_ was calculated to assess the strength of association. Pearson correlation analyses were also conducted in each clinical group to study the relationships between different EF domains and AR and explicit and applied competence in ToM. Because the age differences were close to statistical significance, analyses were performed, introducing age as covariable in the MANCOVA and conducting partial correlations; however, essentially the same results emerged when the analyses were conducted with the covariate.

### Comparison between children with HFASD, ADHD, and TD in social cognition

The MANOVA conducted to assess the main group effect on the conceptual knowledge (NEPSY subscales) and applied knowledge (ToMI) indicators was statistically significant [Wilk's Lambda (Λ) = 0.26, *F*_(16, 232)_ = 13.76, *p* < 0.001, η^2^_*p*_ = 0.48]. ANOVAs showed significant differences in the NEPSY subscales: Affect recognition, *F*_(2, 123)_ = 18.57, *p* < 0.001, η^2^_*p*_ = 0.23; Verbal-ToM, *F*_(2, 123)_ = 13.91, *p* < 0.001, η^2^_*p*_ = 0.18; Contextual-ToM, *F*_(2, 123)_ = 5.84, *p* = 0.004, η^2^_*p*_ = 0.08, and Total-ToM score, *F*_(2, 123)_ = 17.28, *p* < 0.001, η^2^_*p*_ = 0.22. Statistically significant differences were also shown in the applied knowledge task (TOMI): Early-ToMI, *F*_(2, 123)_ = 35.65, *p* < 0.001, η^2^_*p*_ = 0.36; Basic-ToMI, *F*_(2, 123)_ = 64.25, *p* < 0.001, η^2^_*p*_ = 0.51; Advanced-ToMI, *F*_(2, 123)_ = 116.04, *p* < 0.001, η^2^_*p*_ = 0.65 and in the ToMI total score [*F*_(2, 123)_ = 96.42, *p* < 0.001, η^2^_*p*_ = 0.61].

Bonferroni *post-hoc* analyses showed statistically significant differences between the TD group and both clinical groups on AR, Verbal-ToM, and Total-ToM (*p* < 0.001), with no significant differences between clinical groups. On the contextual task, the HFASD group showed significantly worse performance than the TD group (*p* < 0.001), and performance of the ADHD and TD groups was similar. However, there were no significant differences between clinical groups on this task. A different pattern was observed for ToM applied knowledge, where there were statistically significant differences among the three groups, HFASD, ADHD, and TD, on the three subscales of the ToMI and on the Total score (*p* < 0.001). The HFASD group had the lowest score, whereas the TD group had the highest, according to parent estimations. See Table 2.

**Table 2:** Differences between children with ASD, ADHD, and TD on ToM tasks and ToMI Inventory (ANOVAs).

	**TD (*n* = 39)**		**ASD (*n* = 52)**		**ADHD (*n* = 35)**				
	***M***	***SD***	***M***	***SD***	***M***	***SD***	***F*_(2, 123)_**	**η^2^*_*P*_***	**Group differences**
AR	28.36	2.69	23.75	4.09	24.11	4.38	18.57^*^	0.23	ASD, ADHD < TD
ToM V	17.35	2.07	14.01	3.63	15.22	2.79	13.91^*^	0.18	ASD, ADHD < TD
ToM C	4.89	0.96	3.95	1.57	4.34	1.18	5.84^*^	0.08	ASD < TD
ToM Tot	22.25	2.42	17.96	4.15	19.57	3.27	17.28^*^	0.22	ASD, ADHD < TD
ToMI E	18.87	1.22	14.52	3.38	17.22	1.83	35.65^*^	0.36	ASD < ADHD < TD
ToMI B	18.87	1.30	12.92	3.34	16.68	2.14	64.25^*^	0.51	ASD < ADHD < TD
ToMI A	17.00	2.51	8.02	3.01	14.13	3.05	116.04^*^	0.65	ASD < ADHD < TD
ToMI Tot	18.20	1.58	11.50	2.88	15.87	2.13	96.42^*^	0.61	ASD < ADHD < TD

*AR, Affect recognition; ToM V, Theory of mind-Verbal; ToM C, Theory of mind- Contextual; ToM Tot, Theory of mind- Total; ToMI E, ToMI-Early; ToMI B, ToMI Basic; ToMI A, ToMI Advanced*.

* *p < 0.006 (Bonferroni correction)*.

### Relations between EF and social cognition in HFASD and ADHD groups

In the ADHD group, AR significantly correlated with Inhibit (*p* < 0.001), Shift (*p* < 0.001), Emotional control (*p* = 0.012), and the BRI (*p* < 0.001). There were also significant correlations between AR and Monitor (*p* = 0.016). There were no significant correlations between AR and EF in the HFASD group, as Table 3 shows. In the HFASD group, there were significant correlations between the metacognitive component of Initiate and Verbal-ToM (*p* = 0.009) and Total-ToM (*p* = 0.020), both from the NEPSY-II battery. Similarly, there were significant correlations between Initiate and Basic-ToMI (*p* = 0.033), Advanced-ToMI (*p* = 0.046), and Total-ToMI (*p* = 0.040). There were also significant correlations between Organization of materials and Basic-ToMI (*p* = 0.033), advanced-ToMI (*p* = 0.030), and Total-ToMI (*p* = 0.016), and between MI and advanced-ToMI (*p* = 0.043) and Total-ToMI (*p* = 0.026).

**Table 3:** Pearson correlation coefficients between executive functions and social cognition measures in ASD and ADHD groups.

		**AR**	**ToMV**	**ToMC**	**ToMTot**	**ToMIE**	**ToMIB**	**ToMIA**	**ToMITot**
ASD	Inhibit	0.04	0.17	−0.01	0.20	0.04	0.08	−0.01	−0.04
	Shift	0.10	0.18	0.15	0.20	0.11	0.15	0.06	0.13
	Emotional control	0.05	0.24	−0.01	0.26	0.14	0.16	0.17	0.19
	BRI	0.08	0.23	0.05	0.26	0.11	0.14	0.08	0.13
	Initiate	−0.10	−0.36^**^	−0.01	−0.32^*^	−0.15	−0.30^*^	−0.28^*^	−0.29^*^
	Working memory	−0.15	0.04	−0.14	0.03	−0.14	−0.15	−0.16	−0.16
	Plan	−0.09	0.07	0.01	0.06	−0.25	−0.18	−0.26	−0.24
	O. Materials	−0.13	−0.05	−0.11	−0.06	−0.26	−0.30^*^	−0.30^*^	−0.33^*^
	Monitor	0.05	−0.02	−0.02	0.01	−0.19	0.18	−0.17	−0.21
	MI	−0.10	−0.05	−0.07	−0.04	−0.26	−0.26	−0.28^*^	−0.31^*^
ADHD	Inhibit	−0.57^**^	0.07	−0.05	0.03	−0.36^*^	−0.51^**^	−0.17	−0.37^*^
	Shift	−0.47^**^	0.08	0.11	0.15	−0.27	−0.16	−0.07	−0.13
	Emotional control	−0.42^*^	−0.07	−0.03	−0.08	−0.34^*^	−0.29	−0.18	−0.27
	BRI	−0.61^**^	0.03	0.01	0.03	−0.41^*^	−0.42^*^	−0.18	−0.34^*^
	Initiate	−0.06	0.02	0.08	0.15	−0.33	−0.08	−0.19	−0.17
	Working memory	−0.04	−0.03	−0.16	0.01	−0.06	−0.02	−0.03	−0.06
	Plan	0.10	0.25	0.11	0.32	−0.11	0.07	0.11	0.06
	O. Materials	−0.25	0.11	0.01	0.15	−0.38^*^	−0.42^*^	−0.10	−0.34^*^
	Monitor	−0.40^*^	0.03	0.12	0.08	−0.40^*^	−0.41^*^	−0.26	−0.39^*^
	MI	−0.15	0.09	0.02	0.17	−0.30	−0.21	−0.09	−0.21

*AR, Affect recognition; ToM V, Theory of mind-Verbal; ToM C, Theory of mind-Contextual; ToM Tot, Theory of mind-Total; ToMI E, ToMI-Early; ToMI B, ToMI Basic; ToMI A, ToMI Advanced; O. Materials, Organization of Materials; BRI, Behavioral Regulation Index; MI, Metacognition Index*.

* p < 0.05;

** *p < 0.01*.

Correlation analyses in the ADHD group did not show significant associations between ToM tasks on the NEPSY-II and BRIEF subscales. However, there were significant negative correlations between inhibit and Early-ToMI (*p* = 0.034), Basic-ToMI (*p* < 0.001), and total-ToMI (*p* = 0.027), as well as between emotional control and Early-ToMI (*p* = 0.045). The same pattern was observed between the BRI and the Early-ToMI (*p* = 0.014), Basic-ToMI (*p* = 0.013), and Total-ToMI (*p* = 0.049). Regarding the metacognitive processes, there were statistically significant correlations between Organization of materials and Early-ToMI (*p* = 0.026), Basic-ToMI (*p* = 0.012), and Total-ToMI (*p* = 0.049), as well as between Monitor and Early-ToMI (*p* = 0.018), Basic-ToMI (*p* = 0.013), and Total-ToMI (*p* = 0.022).

## Discussion

Research on poor social functioning in children with ASD and ADHD has increased exponentially, suggesting a significant clinical overlap between these two disorders in terms of impairments in social skills. Although, the consequences of social impairment in the trajectory of both disorders is no longer disputed, only a small body of research has examined social cognition deficits comparing groups of children with ASD, ADHD, and TD. The literature review on this area of study reveals two important issues. First, there is a lack of consistency in the findings related to the profile of ToM skills in individuals with ASD and ADHD. Second, another question that remains unresolved is related to the possible functional link between ToM and EF in ASD and ADHD.

Therefore, the first objective of this study was to analyze two fundamental social cognition abilities, affect recognition and ToM, in children with HFASD, ADHD, and typical development, matched on age, IQ, and semantic knowledge. Children with HFASD were expected to have worse performance on all the social cognition measures than children with TD and children with ADHD. The analysis revealed that children with HFASD and children with ADHD, compared to a group of typically developing peers, showed impairments on all the social cognition measures. In addition, consistency is observed in the results, regardless of the ToM assessment methodology employed, that is, performance tasks or parent estimates of daily life behavior. This finding, which is consistent with the initial hypothesis, supports the idea that children with these two disorders experience deficits in AR (Downs and Smith, 2004; Sinzig et al., 2008; Demurie et al., 2011; Demopoulos et al., 2013; Baribeau et al., 2015) and attribution of mental states, such as feelings, beliefs, intentions, and desires (Buitelaar et al., 1999; Adachi et al., 2004; Demurie et al., 2011; Demopoulos et al., 2013; Hutchins et al., 2016).

The use of ToM measures with different levels of complexity, i.e., laboratory tasks of explicit knowledge and applied abilities in everyday life contexts, adds specific nuances to the findings. First, when comparing the ability to recognize basic emotions such as happiness, sadness, fear, anger, and disgust, the performance of children with HFASD and children with ADHD is not significantly different. The ease of distinguishing between different faces is based on holistic processing, that is, the perception of faces as more than the sum of their parts. It is likely that failures of individuals with ASD and ADHD in understanding and interpreting facial emotions are due to the use of predominantly non-holistic, chaotic, and disorganized strategies. They focus attention on some minor facial feature that keeps them from fully understanding someone else's emotional expression. In fact, Berggren et al. (2016) demonstrated that attention had a significant influence on AR in individuals with ADHD and ASD. Furthermore, according with the Weak Coherence Account (Happé and Frith, 2006), individuals with ASD have a perceptual bias for local, rather than global, stimulus features. However, according to a study by Booth and Happé (2010), individuals with ADHD, in spite of their inhibitory deficits, do not have central coherence problems. Consequently, impaired visual processing related to attentional or monitoring strategy deficits might contribute more to the emotion recognition problems observed in the ADHD group than weak central coherence. However, our study cannot provide an answer to this question, which remains open to future research.

On the NEPSY-II ToM verbal subtest, both the HFASD and ADHD groups differed from TD, but not from each other. Both clinical groups were equally affected in their ability to understand first- and second-order false beliefs, double deception, and figurative language, all aspects assessed on the ToM verbal subtest. The HFASD group also presented difficulties on the contextual task, obtaining significantly worse results than the TD group. By contrast, the ADHD group's performance was similar to that of the TD group, suggesting that children with ADHD do not have problems with inferring feelings or mental states of other children based on the visual or physical social context. ADHD children seem to frame emotions based on context clues with greater ease than children with ASD. These results might be related to the “context blindness” hypothesis (Vermeulen, 2014), which states that individuals with ASD experience impairments in the spontaneous use of context in information processing, in this case, of a social and emotional nature.

A different pattern was observed in the applied ToM skills, distinguishing children with HFASD, ADHD, and typically developing peers. Statistically significant differences among the three groups were found on all three ToMI subscales (Early, Basic, and Advanced): Parents perceived that the ASD group had more difficulty than the other two groups in understanding basic emotions, distinguishing between physical and mental, making second-order inferences, or making complex social judgments. In addition, there appeared to be a symptomatic continuity, such that the ADHD group was found in an intermediate position between TD and ASD in the practical application of ToM abilities. The results agree with the trend found in previous work (Demurie et al., 2011; Demopoulos et al., 2013; Baribeau et al., 2015). It is possible that daily life situations estimated by parents require more resources than laboratory executive tasks. In other words, the individuals would be less likely to efficaciously apply their conceptual capacity in real life interactions due to difficulties in parallel processing and in selecting appropriate strategies for the context. However, in any case, real-world, everyday ToM impairments observed by parents or teachers would not reduce the importance of neuropsychological tasks. Instead, they could contribute to a broader understanding of structured performance-based TOM deficits found in the clinical or laboratory setting.

When the achievement levels of children with HFASD and ADHD are compared on the two types of ToM tasks, explicit and applied, the results are not fully consistent with the work of Hutchins et al. (2016). In the only study that applied both types of measures, Hutchins et al. (2016) found “impaired explicit and applied ToM in children with ASD, but intact explicit and impaired applied ToM competence in children with ADHD” (p. 103). Our work coincides with this generalized profile of the ASD group's deficits in mental abilities, regardless of their explicit or applied nature. However, the group with ADHD, in our case, showed deficits on the verbal task of explicit knowledge. One reason the performance of children with ADHD on this task would be equal to that of children with ASD could be a high rate of comorbid learning disabilities. In fact, although we have no objective data, more than 90% of the children with ADHD were receiving educational support for their specific needs at school.

Our results suggest the existence of more severe impairments in social cognition in the HFASD group compared to the ADHD group, although both neurodevelopmental disorders involve difficulties in processing social information. Furthermore, deficits are found on the AR task, which requires perceptual skills, as the children do not have any other information apart from what is contained in the facial expressions, and on the ToM tasks, which require higher thought processes such as logical skills, pragmatic language, and the recognition that one's mind is different from the minds of others. Deficits are also shown in both structured ToM test situations and multidimensional real-life situations, which require more complex and sophisticated skills. Moreover, the worse performance of the group with HFASD on ToM abilities, compared to the group with ADHD, does not seem to be due to language limitations, considering that the two groups were matched on expressive vocabulary. HFASD difficulties may be linked to a more severe meta-representational deficit, an idea that future research will have to verify.

The second objective of this study was to explore the interplay between AR and ToM abilities and “every day” EF in children with HFASD and children with ADHD to find out whether ToM deficits are related to different EF subdomains in these children. A first analysis showed that, in both groups, significant relationships were found on scales measuring the application of ToM skills in the real world. Social interactions in daily life where we must respond spontaneously to a variety of events require more EF resources than laboratory tasks performed in contexts with greater control and stimuli isolation. Hence, it is important to complement the evaluation of ToM performance tasks by measuring how children cope with real-world social interactions that can result in success or failure and have emotional consequences.

As expected, the interrelationships between EF and ToM abilities presented a different profile in ASD and ADHD. In children with ADHD, Inhibit, Shift, Emotional control, and the Behavioral regulation index presented high and significant correlations with AR and Early TOMI. Inhibitory control and the Behavioral regulation index also correlated significantly with the Basic TOMI. Finally, Monitor presented correlations with AR and Early and Basic TOMI scales, underlining the ongoing control that the integration of emotional cues requires in the constantly changing social environment. The findings coincide with previous literature (Yang et al., 2009; Mary et al., 2016) demonstrating a close association in ADHD between inhibition and ToM measures that require ignoring the knowledge we have about a situation and responding from another person's perspective. The overall pattern fits Barkley model (1997), which explains how the involvement of inhibitory control in different EF components (e.g., affect regulation and motivation) affects a wide range of behaviors linked to social information processing.

The correlations in the ASD group show a quantitatively and qualitatively different situation. This group shows a somewhat worse profile that focuses on the EF of Initiate. A poor decisive attitude, or difficulty in thinking of alternatives to solve problems, correlated negatively with performance on the verbal ToM task and, especially, with two scales that assess the application of ToM abilities in the context of everyday life, that is, Basic and Advanced TOMI. Organization of materials was significantly associated with the Basic TOMI, whereas the Metacognitive index was associated with the Advanced TOMI. Previous studies have demonstrated a link between ToM and EF in children and adolescents with ASD, specifically on set-shifting skills (Joseph and Tager–Flusberg, 2004; Pellicano, 2007). Likewise, measures of divided attention and semantic fluency have been significantly associated with social and communication autism symptoms (Kenworthy et al., 2009). In our study, the initiate behavior skills in ASD focus the relationship between EF and ToM on metacognitive subdomains related to the ability to actively solve problems in a variety of contexts. As in previous studies (Leung et al., 2016; Freeman et al., 2017), we found significant associations between metacognitive executive function and social cognition measures in ASD.

Our findings add to existing knowledge about deficits of children with ADHD and HFASD in AR and understanding others' mental states, which reflects a certain symptomatological continuum where HFASD shows greater severity. However, the social impairments of these two neurodevelopmental disorders cannot be explained solely on the basis of ToM failures. According to a recent meta-analysis (Imuta et al., 2016), although performance on ToM tasks is associated with different subtypes of prosocial behavior (helping, cooperating, comforting), the magnitude of the association is relatively weak, and it is still necessary to explore additional paths. Social cognition development is dependent on the maturing of several brain systems, and it is urgent to develop new research to study emotional dysfunctions in ADHD, using not only behavioral measures, but also cerebral activity measures (Albert et al., 2008). Fronto-striatal system dysfunction, an etiology shared by ASD and ADHD (Gargaro et al., 2011), could be an explanatory factor in social cognition and executive problems. However, the brain's organization is shaped by parenting (attachment type, discipline style, parenting stress, coping strategies, sociodemographic family characteristics, parents' mental health), school (learning environments, instructional methodology, teacher cognitive schemes), and the social macrosystem that determines social norms. This exemplifies the complex interactions that occur between brain development and the (social) environment.

The present study has several methodological limitations. Sample size and the fact that most of the children in the ADHD group have a combined presentation may affect the generalizability of the results. Moreover, the low number of females and the recruitment of participants from families with middle-class income levels may not adequately represent the general population. A high percentage of children with ADHD were medicated. Another limitation is related to the ToM measures used. Although, an effort was made to integrate measures of a different nature, it would have been desirable to also incorporate measures based on direct observation protocols. In addition, the present study relied only on teachers' reports of EF, without including supplemental laboratory performance measures. Future work should combine laboratory and informant-based measures for a more in-depth investigation. In particular, the unexpected lack of significant relationships between working memory and ToM in both the HFASD and ADHD groups may be due, at least in part, to the measurement methodology used. It would have been advisable to use other memory indicators such as performance tests. Furthermore, the results of the correlation analyses have an exploratory nature, due to the many variables that intervene in the analyses. However, the application of the Bonferroni correction in this situation would involve a greater possibility of committing a Type II error and rejecting relationships that may exist. Finally, the cross-sectional design does not allow us to conclude whether the interrelationships between mental abilities and EF follow a different course in ASD and ADHD. A longitudinal research design would make it possible to test the directionality of the influence.

Despite these limitations, this study provides evidence about deficits in social cognition processes in children with ASD and ADHD and the differential role of EF. It supports the importance of incorporating EF measures in the evaluation and treatment of children with these two neurodevelopmental disorders. A better understanding of the individual differences in this interdependence will lead to greater possibilities of carrying out successful interventions. Inhibition and behavioral regulation deficits need special treatment that might differ from interventions for metacognitive deficits. Above all, interventions on social problems will be more successful if they are developed from the point of view of performance, the family, and the school, and introducing multimodal components of EF and ToM, given the relationships between these skills.

## Author contributions

AM, CB, BR, IB, and CC each made substantial contributions to the conception and design of the work, to the acquisition, analysis, or interpretation of data for the work, drafting the work or revising it critically for important intellectual content, final approval of the version to be published, and agreed to be accountable for all aspects of the work in ensuring that questions related to the accuracy or integrity of any part of the work are appropriately investigated and resolved.

### Conflict of interest statement

The authors declare that the research was conducted in the absence of any commercial or financial relationships that could be construed as a potential conflict of interest.
